# Meleney's gangrene: A diagnostic pitfall in the context of lower lumbar hypoesthesia – A case study

**DOI:** 10.1016/j.ijscr.2025.112004

**Published:** 2025-09-27

**Authors:** Alina Shrestha, Kishor Manandhar, Asish Shrestha, Rahul Jha, Bijay Raj Bhatta, Nirmal Shrestha

**Affiliations:** Department of General Surgery, National Academy of Medical Sciences, Kathmandu, Nepal

**Keywords:** Case report, Immunocompetent, Mortality, Debridement, Meleney's gangrene, Perianal abscess

## Abstract

**Introduction:**

Meleney's gangrene is one of the conditions classified under *necrotizing “soft tissue infections”*. This uncommon illness has a high fatality rate and requires immediate diagnosis, aggressive antibiotic therapy, and extensive debridement. The mortality rate associated with Meleney's gangrene is approximately 34 %.

**Case presentation:**

Here we report the case of a 50-year-old immunocompetent woman, with no known comorbidities and no recent surgical history. She was initially diagnosed with a perineal abscess; however, after the infection worsened, she was later diagnosed with Meleney's synergistic gangrene. Immediate extensive debridement was performed, and empirical intravenous antibiotics were administered. Shortly after the procedure, and before regaining consciousness, the patient's condition rapidly deteriorated, and she succumbed to death.

**Discussion:**

Early identification of Meleney's gangrene, followed by prompt treatment with broad-spectrum antibiotics and aggressive surgical debridement, is essential for improving outcomes. However, early diagnosis is often challenging, and considerable uncertainty may complicate the process. A delayed diagnosis of Meleney's gangrene significantly increases the risk of mortality.

**Conclusion:**

This case is reported to highlight how delayed diagnosis and late intervention can result in a fatal outcome in cases of Meleney's gangrene. Effective treatment can be implemented only if early diagnosis of this rare form of gangrene is achieved.

## Highlights

• High Mortality Rate:
  Meleney's gangrene, also known as progressive bacterial synergistic gangrene, has a mortality rate that can be as high as 30-40%, and even higher (up to 90%) in patients with diabetes.
• Delayed Diagnosis:
  The initial presentation of Meleney's gangrene often resembles cellulitis, making early diagnosis challenging. This delay in diagnosis significantly impacts mortality.
• Importance of Early Aggressive Treatment:
  Prompt surgical debridement, combined with broad-spectrum antibiotic therapy, is essential for managing this infection and improving patient survival.
• Successful Outcomes with Aggressive Management:
  Case reports have shown that aggressive debridement, along with appropriate antibiotic therapy and supportive care, can lead to successful outcomes, even in severe case.
  The rapid progression and severity of Meleney's gangrene necessitate its recognition and timely intervention.
  Management is centered on a rapid and aggressive multidisciplinary approach.

## 1 Introduction

Meleney's gangrene is a type of necrotizing fasciitis, also known as progressive bacterial synergistic gangrene. Its severity is believed to result from the synergistic effects of multiple bacteria [1], and it has been observed to exhibit the cultural characteristics of a symbiotic organism [2]. Both Fournier's gangrene and Meleney's gangrene are types of necrotizing fasciitis, as they share the characteristics of aggressive life-threatening conditions. Their primary distinction lies in their typical location and the specific bacteria involved. Fournier's gangrene primarily affects the genital and perineal regions and is usually a polymicrobial infection, often originating from the gastrointestinal or genitourinary tracts. Meleney's gangrene usually manifests following surgery or even after minor trauma [2], and the infection spreads rapidly, involving the skin, subcutaneous tissue, and in severe cases, the deep fascia as well.

The ulcers that form at the center of the lesion are usually covered by a black eschar and surrounded by a gangrenous margin. First described by Dr. Meleney and Dr. Brewer in 1926, its microbial effectors were further classified by Meleney in 1931 [3]. Due to its rarity and high mortality rate, this infection needs to be diagnosed promptly and treated aggressively with antibiotics and rigorous debridement [4]. We are reporting this case because, in rural areas with limited healthcare facilities, the prognosis of this disease is often poor. By documenting the specifics of the case—including symptoms, disease progression, and patient history—this report may contribute to the collective understanding of how this condition manifests.

This work has been reported in line with revised SCARE criteria, 2025 [5].

## 2 Case presentation

A 50-year-old woman with no medical comorbidities presented with complaints of pain, discharge, and swelling in the perianal region for 7 days, followed by redness over the abdominal skin for 4 days. She also reported a low-grade fever for 3 days. The patient had decreased sensation below the lumbar region following excision of a myelocele approximately 25 yrs. ago. Except for a slightly limping gait, her daily activities were unaffected. Initially, she was managed at a local hospital as a case of perianal abscess with oral antibiotics, but after the development of skin changes over the lower anterior abdominal wall, she was referred to our center. She arrived to our center after 7 days of developing symptoms.

**Fig. 1:**
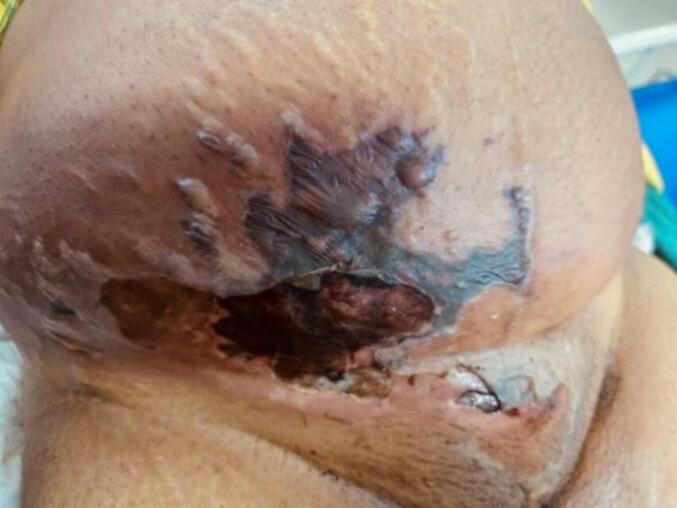
Presentation features: ruptured blisters, edematous, gangrenous and necrosed skin.

On presentation she was tachypneic and tachycardic with hypotension. The skin over the perineal and perianal regions was edematous, with multiple serous fluid-discharging openings in the perianal area. The skin over the right lower abdomen was reddened, warm, tender, and indurated (Fig. 1). There was visible bullae and skin was necrosed. On palpation over affected area crepitus was felt. Remaining areas of the abdomen were soft and non-tender. In the perineal region, the wound was limited with minimal tissue give away, whereas in the perianal region, there were sinuses draining pus. These findings guided us to classify this case as Meleney's gangrene rather than Fournier's gangrene. The wound could be tracked from the perineum to anterior abdominal wall, indicating that the infection had spread from a perianal abscess. Per vaginal and per rectal examinations were essentially normal. The initial total leukocyte count was 11,000/cm^3^, and the remaining baseline laboratory investigations were within normal limits. She was started on intravenous empirical antibiotics; and emergency debridement was done under general anesthesia within an hour of admission.

**Fig. 2:**
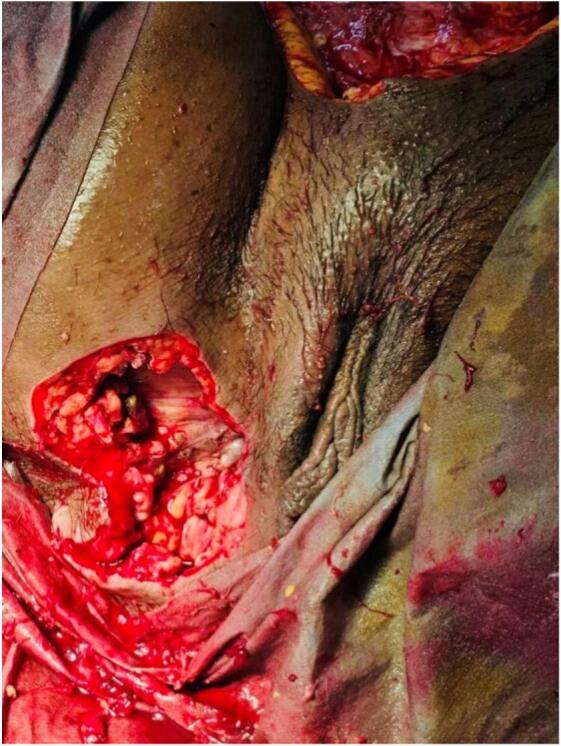
Perineal wound communicating with abdominal wound.

**Fig. 3:**
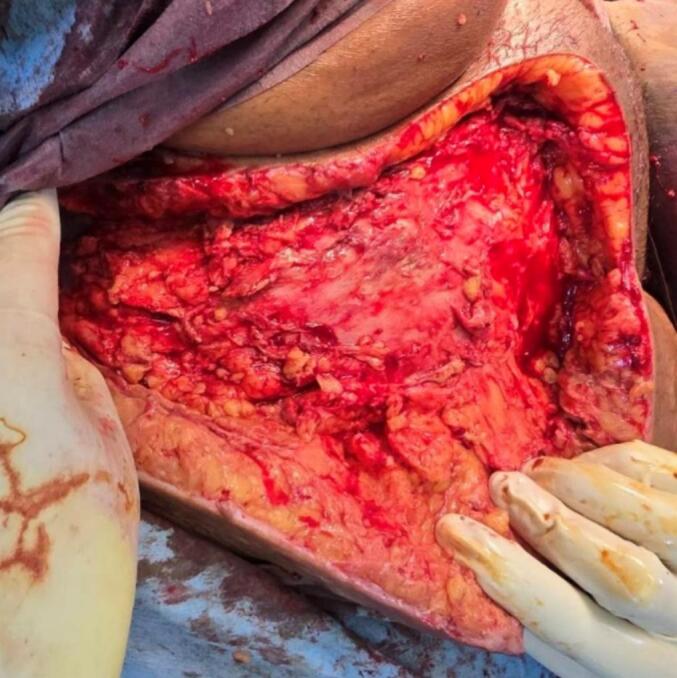
Intra operative picture of extensive debridement of anterior abdominal wall.

Debridement involved the removal of all gangrenous tissue, slough and devitalized tissue until healthy margins were visible. Tissue samples were sent for culture and sensitivity testing. The wound was thoroughly debrided using hydrogen peroxide and washed with normal saline. Hemostasis was maintained, and the wound was packed with sterile wet dressing. Intraoperatively, a communication tract was observed between perineal region and anterior abdominal wall (Fig. 2). The tissues over the lower anterior abdominal wall were necrosed, with dish water collection. The subcutaneous tissue of the right iliac region was extensively involved, sparing the fascial layer (Fig. 3). Intraoperatively, the patient required inotropic support to maintain her blood pressure and couldn't be extubated. She was transferred directly to the ICU direct from the operation theatre. Post-operatively, her mean arterial pressure was not maintained, necessitating the initiation of triple inotropic.

Support, and her Total Leucocyte Count rose to 27,000/cm^3^. Despite debridement and intensive supportive care—including intravenous fluids, inotropes, and antibiotics—her condition continued to deteriorate. Blood pressure and urine output did not improve, and she ultimately succumbed to death secondary to septic shock 48 h postoperatively.

## 3 Discussion

This case highlights the aggressive nature and potential for rapid deterioration associated with Meleney's gangrene. Gangrene refers to the death of body tissue due to lack of blood flow or a severe bacterial infection. Necrotizing fasciitis is characterized by spreading inflammation of the skin, deep fascia, and soft tissues, with extensive tissue destruction and toxemia, commonly caused by mixed infections involving anaerobes, coliforms, and gram-negative organisms [6]. Meleney's gangrene is typically observed in the truncal region [7]. Meleney's gangrene is diagnosed based on a combination of distinctive clinical features, microbiological findings, and histological analysis. Since it is a rare and severe form of necrotizing fasciitis, a high index of suspicion is required for early diagnosis. A series of experiments in animals suggested that the association of the microaerophilic Streptococcus and the *Staphylococcus aureus* is responsible for the lesion and represents an example of bacterial symbiosis or synergism [1]. In this condition, there is extensive inflammation of the subcutaneous soft tissues and skin, leading to rapid tissue destruction, septicemia, and potentially death. The necrotizing process may primarily affect the fascia, resulting in secondary gangrene of the overlying skin due to thrombosis of subcutaneous blood vessels, or it may primarily affect the skin [8].

Diabetes, drug addiction, alcohol, obesity, malnutrition, tumors, immunodeficiency, and other chronic medical conditions are important predisposing factors [9]. The process typically begins in the skin and subcutaneous tissues and then to the fascia; however, some studies have shown that the necrosis can penetrate even deeper [6]. A delayed diagnosis of Meleney's gangrene is associated with a higher risk of mortality. The key triggers for rapid and immediate surgical intervention are indicators of rapid progression and systemic toxicity. Disproportionate pain, Rapidly spreading erythema, Presence of the characteristic “triple zone”, Blistering and bullae formation, Crepitus, Tenderness beyond the visible borders are some clinical indicator that guides urgent intervention. Patient may also develop systemic signs of sepsis like tachycardia, confusion, hypotension. Serial debridement and regular dressing may necessitate an extended hospital stay, and in some cases, skin grafts may be required for the healing of wound with larger raw areas [6]. Early detection and diagnosis of Meleney's gangrene is usually challenging because of its early presentation similar to cellulitis or abscess. Our patient was also initially misdiagnosed and managed as a perianal abscess at another center, which delayed her referral to a higher center. Prompt recognition and management of Meleney's gangrene within the first 24 h can reduce mortality from 70 % to 35 % [9].

Currently, it is recommended that all necrotizing infections be classified as necrotizing soft tissue infections (NSTIs) and managed using standardized diagnostic and treatment protocols. This approach facilitates rapid identification and treatment, both of which are critical to improving outcomes and reducing mortality in patients with NSTIs [10]. In our case, tissue cultures were sent intraoperatively, but the results couldn't be traced. Most reported cases of Meleney's gangrene occur following postoperative wound infection in diabetic patients [11]. Our patient was nondiabetic, but she had decreased sensation to touch and pain below the lumbar region following excision of myelocele, which explains her delayed presentation to hospital. The absence of traditional risk factors, such as recent surgery or diabetes in our patient underscores that this infection can occur in otherwise healthy individual. In this case, the patient underwent extensive debridement with excision of all necrotic tissues and administration of broad-spectrum antibiotics; however, due to the delay in presentation, she could not be saved.

## 4 Conclusion

Surgical debridement combined with timely initiation of broad-spectrum empirical antibiotics remains the cornerstone of treatment for Meleney's gangrene. Delayed presentation significantly increases both morbidity and mortality. In addition to immunocompromised status and uncontrolled diabetes mellitus, decreased pain perception should also be recognized as an important risk factor for Meleney's gangrene.

## Author contribution

1. Constructing hypothesis for the manuscript: Alina Shrestha.
2. Planning methodology to reach the conclusion: Alina Shrestha, Rahul Jha, Bijay Raj Bhatta, Kishor Manandhar, Asish Shrestha, Nirmal Shrestha.
3. Organizing and supervising the course of the article and taking responsibility: Alina Shrestha, Rahul Jha, Bijay Raj Bhatta Kishor Manandhar, Asish Shrestha.
4. Patient follow-up and reporting: Alina Shrestha.
5. Logical interpretation and presentation of the results- Bijay Raj Bhatta, Alina Shrestha, Rahul Jha.
6. Construction of the whole or body of the manuscript- Alina Shrestha.
7. Reviewing the article before submission not only for spelling and grammar but also for its intellectual content- Alina Shrestha, Bijay Raj Bhatta, Kishor Manandhar, Nirmal Shrestha.

## Consent

Written informed consent was obtained from the patient's family member (patient's own son as patient died soon after admission to hospital) for publication of this case report and accompanying images. A copy of the written consent is available for review by the Editor-in-Chief of this journal on request.

## Ethical approval

Ethical approval is waived at our institution (National Academy of Medical Science, Bir Hospital) and this study was exempt from ethical approval at our institution, as this paper reports a single case that emerged during a normal surgical case report.

## Guarantor

Alina Shrestha accepts full responsibility for the work and/or the conduct of the study, has access to the data, and controls the decision to publish.

## Research registration number

Not applicable.

## Funding

None.

## Conflict of interest statement

None.
